# Impacts of amino acid-linked platinum(II) complexes on DNA structure

**DOI:** 10.1007/s00775-025-02097-x

**Published:** 2025-01-24

**Authors:** Deepak Shrestha, Bett Kimutai, Christine S. Chow

**Affiliations:** Department of Chemistry, Wayne State University, Detroit, MI USA

**Keywords:** Cisplatin, Amino acid-linked platinum(II) compounds, Antiproliferative activity, DNA bending

## Abstract

The discovery of cisplatin (cisPt) as an effective anticancer agent was a milestone in the health industry. Despite its success, undesired side effects and acquired resistance still limit the therapeutic usefulness of cisPt. Intrastrand adduct formation at consecutive purines and structural modifications of DNA caused by platinum(II) complexes are important factors for antitumor efficacy. In this study, we examined amino acid-linked platinum(II) complexes, collectively referred to as AAPt, for antiproliferative activity and ability to induce DNA bending. The antiproliferative activity of one AAPt complex tested against a prostate cancer cell line was comparable to that of cisPt, whereas only activity of the AAPt complex was lower in a normal human prostate cell line. Various AAPt analogues were examined for impact on the structures of DNAs with four different purine dinucleotide target sites (GG, AG, GA, and AA) and compared to the parent cisPt. The roles of side-chain identity, chirality, and coordination type (*e.g*., (*N,O*) vs. (*N,N*)) of AAPt complexes are discussed with respect to DNA adduct formation and ability to induce DNA bending. Although the AAPt complexes display different nucleotide preferences (A for AAPt vs. G for cisPt), DNAs containing GG-platinum adducts display a greater degree of bending compared to DNAs with AA-platinum adducts.

**Graphical abstract:**

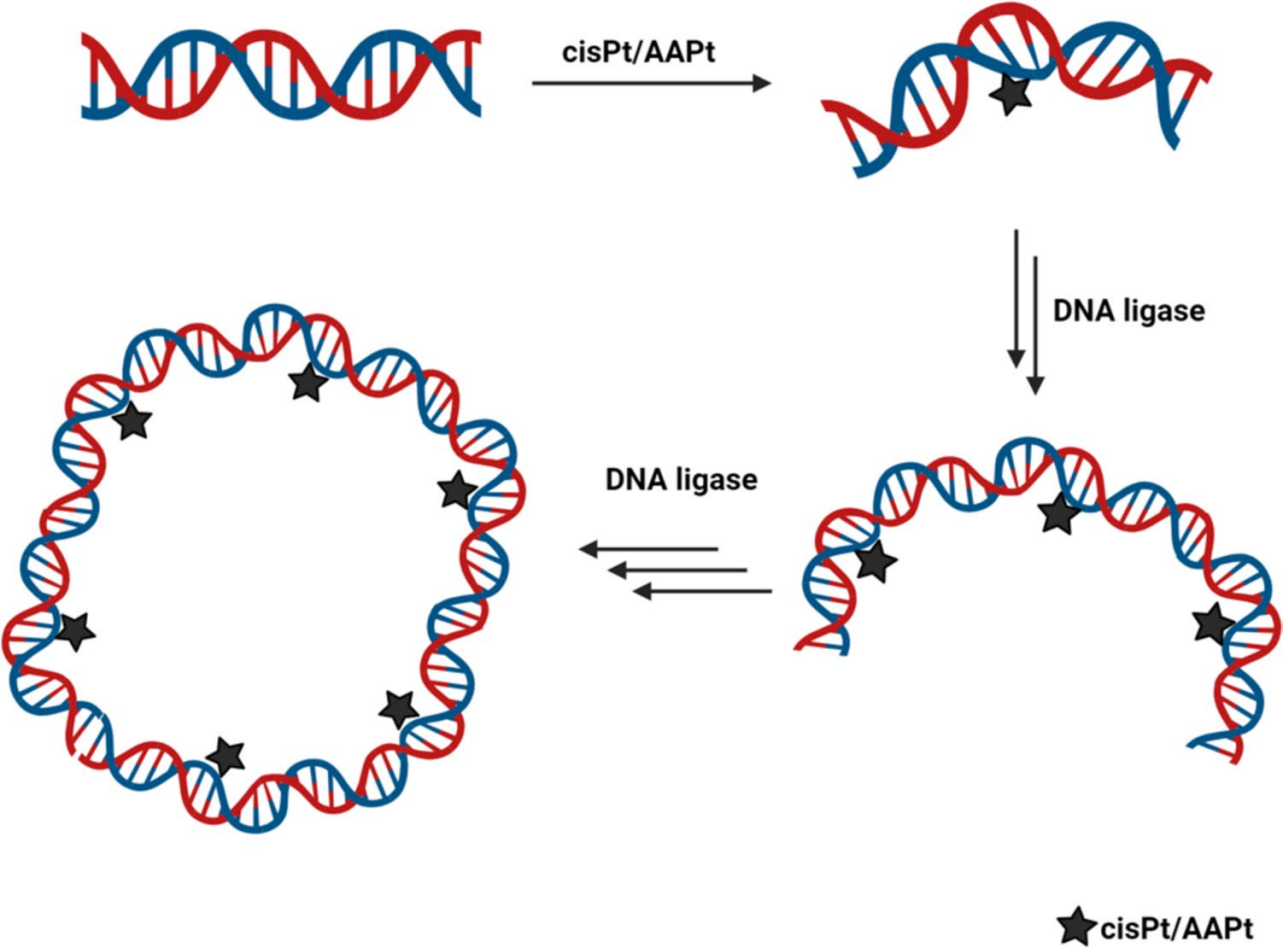

**Supplementary Information:**

The online version contains supplementary material available at 10.1007/s00775-025-02097-x.

## Introduction

**Fig. 1:**
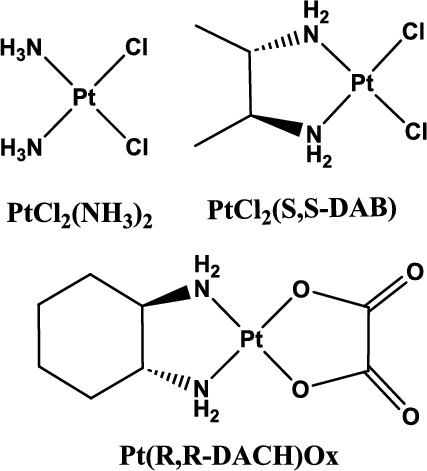
Cisplatin and chiral analogues. Cisplatin [PtCl_2_(NH_3_)_2_] (upper left), [PtCl_2_(*S,S*−2,3-diaminobutane)] (upper right), and oxaliplatin ([Pt(*R,R*−1,2-diaminocyclohexane) (oxalato)]) (lower center) are shown

The metal complex *cis-diamminedichloridoplatinum(II),* or cisplatin (cisPt) (Fig. 1), is used for treatment of a variety of cancers including testicular, ovarian, head and neck, and lung [1] The compound was approved for clinical use in 1978 [2–5]. The anticancer activity of cisPt is attributed to its high reactivity with DNA [6, 7], although reactions can also occur with RNA and proteins [8–10]. Despite its success in treating some forms of cancer, cisPt treatment causes severe side effects, and drug resistance develops in some patients [11] Therefore, analogues with higher efficacy and fewer side effects are still needed. Two platinum-based drugs used clinically are carboplatin and oxaliplatin (Fig. 1) [12, 13]. Many of the platinum analogues generated have varying ligands; however, the impact of alternative ligands on platination kinetics, target selection, adduct type, and DNA structure have not been fully explored [12, 14].

There is much evidence that points to DNA as the main target of cisPt, which in its bisaquated form generates adducts with the DNA nucleobases and inhibits cellular processes such as transcription and replication [2, 8, 15]. More specifically, the bisaquated form of cisPt coordinates at consecutive purines, with G preferred over A residues, to form the major d(GpG) and minor d(ApG) intrastrand adducts [16–19]. The N7 positions of G and A coordinate to the platinum metal center [20], as observed by X-ray crystallography and NMR spectroscopy [19, 21]. The d(GpG) adducts cause conformational changes in the DNA, including a directional bend towards the major groove [16, 18, 19, 22]. Moreover, local unwinding, perturbations of hydrogen bonding at the adduct site, widening and flatting of the DNA minor groove, a change of local helicity from B to A form, and lower thermodynamic stability of the DNA have also been reported [23–25]. The changes in DNA structure caused by cisPt such as bending can attract proteins that normally bind to bent DNA or cause DNA bending [26, 27]. Protein binding to platinated DNA could have negative effects on cellular functions. For example, bound proteins might block the actions of DNA repair proteins or block transcription, leading to cell death [20, 28, 29]. In order to play a role in the anticancer mechanism of cisPt, the proteins would likely have differing roles in normal versus cancer cells [27].

**Fig. 2:**
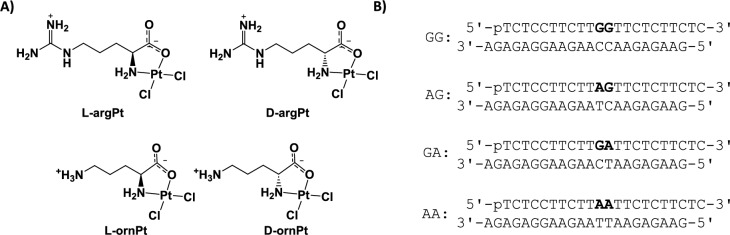
Platinum compounds and DNA sequences. **A** Amino acid-linked platinum(II) complexes (AAPt) used in this study (arg, arginine; orn, ornithine) are shown. **B** Double-stranded DNAs employed in the bending experiments have a phosphorylated top strand with one possible site (d(GpG), d(ApG), d(GpA), or d(ApA)) for 1,2-intrastrand adduct formation with either cisPt or AAPt and a complementary bottom strand with two-nucleotide overhangs on the 3′ ends

Amino acids can be used to generate cisPt analogues (AAPt, Fig. 2). Amino acid ligands provide a good range of diversity with respect to charge, size, hydrophobicity, hydrogen-bonding capabilities, and stereochemistry. The AAPt complexes potentially have varying reactivities with different preferred sites of adduct formation compared to cisPt. The predominate sites of coordination for platinum complexes with ornithine (ornPt) and arginine (argPt) (shown in Fig. 2) are the N7, and N1 or N3 positions of A [30, 31].

Cisplatin analogues with chiral ligands have been synthesized and tested for their biological activity [32–38]. For example, the stereoisomers of [PtCl_2_(*S,S*−2,3-DAB)] (DAB: diaminobutane) and [PtCl_2_(*S,S*−1,2-DACH)] (DACH: diaminocyclohexane) having *S* configuration at the chiral carbons are more active against strains of *Salmonella typhimurium* [32]. The *R,R* stereoisomer of oxaliplatin ([Pt(*R,R*−1,2-DACH)Ox], Ox: oxalato, Fig. 1), which forms DNA adducts similar to cisPt, is approved for clinical use [39, 40]. Oxaliplatin, similar to cisPt, causes bending and unwinding of DNA; however, for oxaliplatin, these effects are dependent on the flanking sequences surrounding the adduct site [40]. The DNA bend angles created by adducts of the stereoisomers [Pt(*R,R*-DACH)Ox] and [Pt(*S,S*-DACH)Ox] are different (31° and 23°, respectively), and these angles also vary with the flanking sequences [40]. Of note, the *R,R* isomer of oxaliplatin, which is approved for clinical use, causes DNA bending to a similar extent as cisPt (~ 31–34°) and exhibits higher anticancer activity than the *S,S* isomer [40]. Similarly, two other examples of stereoisomers are [PtCl_2_(*R,R*−2,3-DAB)] and [PtCl_2_(*S,S*−2,3-DAB)], which form intrastrand cross-links and induce DNA bend angles of 35° and 24°, respectively [37]. These prior studies provide important information regarding the impact of ligand structures (*e.g*., size, charge, hydrophobicity, and chemical composition) and chirality of various platinum complexes on bending of DNA oligonucleotides in a sequence-dependent manner [37, 40, 41].

In the present study, we investigated the structural impacts of DNA intrastrand adducts with the enantiomers (L and D) of two AAPt complexes, ornPt and argPt. Of the two AAPts tested, argPt shows higher potency towards prostate cancer cells with good selectivity over normal cells [42]. We also examined the role of sequence at the adduct site by monitoring the ability of cisPt and AAPt analogues to bend DNA. Oligonucleotides (Fig. 2) containing site-specific 1,2-d(GpG), d(ApG), d(GpA), and d(ApA) platinum adducts were generated and the degree of bending was determined by ligation and gel electrophoretic mobility assays. Prior studies by Koo and Crothers established an empirical relationship between gel mobility and DNA curvature [16, 43, 44]. These relationships were used to quantify the extent of bending of DNAs containing different purine sequences and 1,2-intrastrand AAPt adducts.

## Materials and methods

### Materials

Cisplatin, potassium tetrachloroplatinate (K_2_PtCl_4_), acrylamide, bisacrylamide, *N,N,N′,N′-*tetramethylethylenediamine (TEMED) d-arginine (d-arg), d-ornithine (d-orn), Tris base, urea, ethylenediaminetetraacetic acid (EDTA), and 3-hydroxypicolinic acid (3-HPA) were purchased from Sigma-Aldrich (St. Louis, MO). l-Arginine (l-arg) and l-ornithine (l-orn) were purchased from Alfa Aesar (Haverhill, MA). D_2_O was purchased from Cambridge Isotope Laboratories (Tewksbury, MA). Silver nitrate (AgNO_3_), ammonium acetate (NH_4_OAc), sodium acetate (NaOAc), ethanol, methanol, and boric acid were purchased from Fisher Scientific (Waltham, MA). Both mono and dibasic sodium phosphate (NaH_2_PO_4_, and Na_2_HPO_4_, respectively) were obtained from EMD Millipore (Kankakee, IL). The ^32^P-labeled adenosine triphosphate (ATP) was from Perkin Elmer (Waltham, MA). Enzymes T4 DNA ligase, T4 polynucleotide kinase (PNK), and exonuclease RecBCD were from New England Biolabs (Ipswich, MA). The DNA oligonucleotides used in this study were purchased from Integrated DNA Technologies (IDT, Coralville, IA). The following sequences were employed: phosphorylated top strands with platination sites in bold (GG: 5′-pTCTCCTTCTT**GG**TTCTCTTCTC-3′, AG: 5′-pTCTCCTTCTT**AG**TTCTCTTCTC-3′, GA: 5′-pTCTCCTTCTT**GA**TTCTCTTCTC-3′, and AA: 5′-pTCTCCTTCTT**AA**TTCTCTTCTC-3′); bottom strands with nucleotides complementary to the platination sites in bold (CC: 5′-GAAGAGAA**CC**AAGAAGGAGAGA-3′, CT 5′-GAAGAGAA**CT**AAGAAGGAGAGA-3′ TC: 5′-GAAGAGAA**TC**AAGAAGGAGAGA-3′, and TT: 5′-GAAGAGAA**TT**AAGAAGGAGAGA-3′).

The human cell lines H1299 (lung), MDA-MB-231 (breast), and MDA-MB-453 (breast) were provided by Dr. Young-Hoon Ahn, DU145 (prostate cancer) was obtained from Dr. Kenneth Honn, and RWPE-1 (normal prostate) was provided Dr. Zhihui Qin (Wayne State University). The cell culture supplies Dulbecco’s modified eagle medium (DMEM), keratinocyte serum free medium (K-SFM), human recombinant growth factor 1–53 (EGF 1–53), bovine pituitary extract (BPE), phosphate buffer saline (PBS), trypsin–EDTA (0.25%) were purchased from Gibco-ThermoFisher Scientific (Waltham, MA).

### Synthesis of AAPt

l-/d-OrnPt and l-/d-argPt were synthesized as previously described [45, 46] with some modifications. Briefly, 0.17 mmol (70 mg) of K_2_PtCl_4_ was dissolved in 1 mL of MilliQ deionized water (ddH_2_O) in a 2 mL microcentrifuge tube. In a separate tube, 0.42 mmol (2.5 eq) of amino acid was dissolved in 1 mL of ddH_2_O. The two solutions were combined in a 2 mL centrifuge tube and vortexed briefly. The solution was incubated in a thermomixer at 50 °C and 950 rpm for 15 h. The mixture was centrifuged for 10 min and the supernatant containing the product was transferred to another tube, then frozen and lyophilized to dryness. The resulting solid was yellowish (ornPt) or greenish (argPt) in color. The solid was washed with ice-cold ddH_2_O three times and then dried on a speed vac concentrator. An additional recrystallization step was carried out to remove any additional impurities (*e.g*., black solids). For the recrystallization step, AAPt was dissolved in minimum amount of hot solvent (MilliQ water) and then allowed to cool to room temperature. The solution was kept on ice until crystals formed. The crystals were collected by filtration and dried. The AAPt complexes were characterized by NMR spectroscopy (Figure S1):

Arg: ^1^H NMR (400 MHz, D_2_O) δ 3.66 (t, *J* = 6.1 Hz, 1H), 3.14 (t, *J* = 6.9 Hz, 2H), 1.85–1.76 (m, 2H), 1.70–1.51 (m, 2H). l-argPt: ^1^H NMR (400 MHz, D_2_O) δ 3.51 (q, *J* = 6.0, 5.2 Hz, 1H), 3.16 (t, *J* = 6.5 Hz, 2H), 1.93–1.78 (m, 2H), 1.78–1.61 (m, 2H). ESI-HRMS: m/z calcd for C_6_H_13_Cl_2_N_4_O_2_Pt [M-H]^−^ 438.0062; found 438.0070. d-argPt: ^1^H NMR (400 MHz, D_2_O) δ 3.51 (q, *J* = 6.1, 5.5 Hz, 1H), 3.16 (t, *J* = 6.5 Hz, 2H), 1.93–1.79 (m, 2H), 1.79–1.60 (m, 2H). ESI-HRMS: m/z calcd for C_6_H_13_Cl_2_N_4_O_2_Pt [M-H]^−^ 438.0062; found 438.0071.

Orn: ^1^H NMR (400 MHz, D_2_O) δ 3.68 (t, *J* = 6.0 Hz, 1H), 2.95 (t, *J* = 7.5 Hz, 2H), 1.88–1.76 (m, 2H), 1.76–1.58 (m, 2H). l-ornPt: ^1^H NMR (400 MHz, D_2_O) δ 3.54 (q, *J* = 5.6 Hz, 1H), 2.97 (t, *J* = 7.2 Hz, 2H), 1.93 (m, *J* = 21.1, 15.2, 11.0, 6.0 Hz, 2H), 1.77 (m, *J* = 21.8, 12.0, 6.6 Hz, 2H ESI-HRMS: m/z calcd for C_5_H_11_Cl_2_N_2_O_2_Pt [M-H]^−^ 395.9844; found 398.9852. d-ornPt: ^1^H NMR (400 MHz, D_2_O) δ 3.54 (q, *J* = 5.4 Hz, 1H), 2.97 (t, *J* = 7.3 Hz, 2H), 2.00–1.85 (m, 2H), 1.76 (m, *J* = 19.3, 10.3, 6.5 Hz, 2H). ESI-HRMS: m/z calcd for C_5_H_11_Cl_2_N_2_O_2_Pt [M-H]^−^ 395.9844; found 398.9851.

### Bisaquation of cisPt/AAPt

For cisPt/AAPt, bisaquation (referred to as the “activation” step, in which the two chlorido ligands are replaced with two H_2_O ligands) was carried out prior to reacting with DNA. A 1:2 molar ratio of cisPt/AAPt and AgNO_3_ (typically 2.5:5 μmoles) in 1 mL H_2_O was vortexed overnight in the dark. After ~ 14 h, the cloudy, white solution was centrifuged to precipitate the AgCl; the supernatant was removed and centrifuged again, with three repeats. The freshly prepared solution consisting of activated cisPt/AAPt was used immediately for reactions with the DNA oligonucleotides.

### Platination of DNA oligonucleotides

Each 22-nucleotide (nt) single-stranded DNA (top strand) with GG, AG, GA, or AA in the middle of the strand was platinated with bisaquated cisPt or one of the four AAPt analogues. For the platination step, 10 nmol of DNA was reacted with 20 nmol of bisaquated cisPt/AAPt in 10 mM phosphate buffer at pH 6.4 overnight (approximately 14 h). The reaction mixtures were mixed with urea to a final concentration of 7 M, placed in a boiling water bath for 2 min, cooled on ice for 2 min, and loaded onto 20% denaturing polyacrylamide gels (20:1 acrylamide:bisacrylamide). The bands corresponding to the platinated (slower mobility) and unplatinated (faster mobility) DNAs were visualized by UV shadowing on a fluorescent TLC plate. The platinated DNAs were excised from the gel and isolated by the “crush-and-soak” method [47] with 350 mM NaOAc or NH_4_OAc buffer and 1 mM EDTA. The DNA-containing solution was filtered through a polyprep column (Bio-Rad, Hercules, CA) and then desalted on a Sep-pak (Waters, Milford, MA) column. The platinated DNAs containing single cisPt or AAPt adducts were characterized by matrix-assisted laser desorption/ionization time-of-flight mass spectrometry (MALDI-TOF MS).

### Characterization of platinated DNAs by MALDI-TOF MS

A supersaturated solution of the MALDI matrix 3-hydroxypicolinic acid (3-HPA) was prepared in 1:1 acetonitrile water. The unplatinated DNA control or platinated DNAs were isolated by the crush-and-soak method as described above with NH_4_OAc buffer followed by Sep-pak desalting. To further desalt the DNA samples, ethanol precipitation was done with NH_4_OAc and the DNA pellets were washed with 80% ice cold ethanol as described previously [48] The desalted DNA samples were dried on a speed vac and then redissolved in ddH_2_O. Approximately 30–40 pmol of the purified, desalted DNA was mixed well with 1 µL of saturated 3-HPA and spotted on a MALDI plate (MTP 384 target plate). The spotted samples were left to dry for 1 h and the masses were obtained on a Bruker UltrafleXtreme MALDI mass spectrometer.

### Radiolabeling, ligation, and gel electrophoresis of DNA oligonucleotides

The bottom-strand oligonucleotides were radiolabeled at the 5′ ends with T4 PNK following the New England Biolabs protocol [49, 50]. The platinated top-strand DNAs and the complementary bottom strands (^32^P labeled at the 5′ end) were annealed. For the annealing step, 20 pmol of platinated DNA and 100,000–200,000 CPM of the complementary strand were mixed in a microcentrifuge tube, heated in a boiling water bath, slowly cooled to room temperature in the bath (typically 12 h), and further cooled to 4 °C for one h. The annealed DNAs were ligated by the following method: the annealed samples were mixed 1/10th volume DNA ligase buffer and 100 U of T4 DNA ligase, and incubated for one h at 37 °C. The ligated DNAs were mixed with 1/10th volume glycerol and loaded onto 8% native polyacrylamide gels (29:1 acrylamide:bisacrylamide). The gel was exposed overnight to a phosphorimager screen and visualized using a Typhoon™ FLA9500.

### Quantification of platinum-195 in cell lines

Platinum-195 quantification was performed using inductively coupled plasma mass spectrometry (ICP-MS) on an Agilent 7700X equipped with an ASX-500 series autosampler and Agilent MassHunter software (Santa Clara, CA). The human prostate cancer cell line DU145 and normal prostate cell line RWPE-1 were grown in 100 × 20 mm tissue culture dishes until confluency. The cells were detached from the bottom of the dishes by treating with trypsin. The suspension was homogenized, and the cell number was determined using a hemocytometer. From the stock suspension, 460,000 cells were seeded on 60 × 15 mm culture dishes each and grown in an incubator (37 °C, 5% CO_2_) for 18 h. The media was removed, and the dishes were washed with 3 mL PBS. In each dish, 3 mL of keratinocyte media with 50 µM of platinum compound was added. The dishes were incubated for another 2 h to allow uptake of the compounds by the attached cells. The media was removed, and dishes were washed again with 3 mL of PBS. The cells on the dishes were resuspended using 500 µL of trypsin and collected into centrifuge tubes. The cell number in each tube was counted using a hemocytometer. The cells were chemically digested using 300 µL of concentrated HNO_3_. The samples were left to further digest at room temperature for 16 h after which they were diluted by adding 2.7 mL of 2% HNO_3_ and 0.5% HCl (in ddH_2_O) solution prior to ICP-MS quantification. For ICP-MS calibration, a platinum standard series with 0.5, 1, 5, 10, 25, 50, 100, 200 ppb concentrations was prepared in 2% HNO_3_ and 0.5% HCl (in ddH_2_O). Aside from the standards and the digested cell samples, solvent blanks and blanks without platinum compounds were also included in the analysis. Quantification was carried on the mass spectrometer with plasma gas flow 15 L/min, nebulizer pump speed 0.3 rps, spectrum acquisition mode, and 100 sweeps per sample, with three replicates per sample and general-purpose plasma 62 mode. A 10-ppb concentration of ^209^Bi solution was used as the instrument internal standard. The ^195^Pt isotope was used for detection with minimal polyatomic interference [51]. Data were collected under the high energy He (HEHe) tune mode in which the internal standard signal was stable and the R value for the calibration curve was closest to 1. For quality control, a 10-ppb standard was run after the samples.

### MTT assays with human cell lines

The number of cells was determined using a hemocytometer. The cells (and media) were seeded in a transparent flat-bottom, 96-well plate with each well having 10,000 cells. The seeded plate was placed in an incubator (37 °C, 5% CO_2_) for 24 h to allow the cells to attach to the wells. Afterwards, the media was removed and fresh media with varying concentrations of the AAPt compound or cisPt added to the wells. In each plate, two types of controls were included. The first, referred to as the growth control, contained cells and media but no platinum compounds. The second, referred to as the blank control, had media only. The plates were incubated for 72 h after which 10 µL of MTT stock (5 mg/mL) was added to each of the wells. The plates were incubated for another 4 h during which time insoluble formazan was produced by viable cells. DMSO (100 µL) was added to the wells and incubated for 10 min to dissolve the formazan. The optical density (OD) of each well was measured at 520 nm using a microplate reader, BioTek Synergy H1 hybrid reader.

### Identification of circular DNA

The ligated DNA samples were mixed with 20 U exonuclease RecBCD with the corresponding buffer from New England Biolabs and incubated for 20 min. The enzyme cleaves linear single-/double-stranded DNA, leaving circular DNAs intact. The reaction mixture was loaded onto 8% native polyacrylamide gels (29:1 acrylamide: bisacrylamide) along with ligated samples that were not treated with exonuclease.

## Results and discussion

### Antiproliferative activity and cellular accumulation of platinum(II) complexes

Cisplatin, or cisPt, is employed for treatment of various cancers, but its use is still limited due to resistance, toxicity, and reduced accumulation [52–54]. Second generation compounds such as oxaliplatin have reduced toxicity and resistance but also have lower potency compared to cisPt [55, 56]. In the search for compounds with a better balance between potency, selectivity (*i.e*., cancer vs. normal cells), and uptake, two AAPt complexes, l-ornPt and l-argPt, were examined for antiproliferative activity in human cell lines. The half maximal inhibitory concentration (IC_50_) values for the AAPt compounds with several cancer (breast, lung, and prostate) and normal (prostate) cell lines were determined using an MTT assay [57]. More specifically, concentrations resulting in 50% reduction of viable cells (IC_50_ values) at 72 h were determined.

The prostate cancer cell line DU145 was tested first because previous researchers reported an IC_50_ value of 4 µM with the parent cisPt [58]. We obtained the same IC_50_ value of 4 µM for cisPt (Table 1), whereas l-ornPt and l-argPt are 12-fold and three-fold less potent, respectively. The more active l-argPt was further tested in lung and breast cancer cells, resulting in three- to four-fold higher IC_50_ values compared to cisPt, consistent with prior studies [45]. Despite its lower activity in the chosen cancer cell lines, l-argPt was found to be much less toxic to normal prostate cancer cells RWPE-1 (IC_50_ 69 µM; six-fold difference with DU145) compared to cisPt that has identical activity in DU145 and RWPE-1 cells (IC_50_ 4 µM).

**Table 1:** Comparison of IC_50_ values of cisPt and AAPt in cancer (DU145 prostate, MDA-MB-231 breast, MDA-MB-453 breast, H1299 lung) and normal (RWPE-1 prostate) cell lines

Compound	Cell line^ref^	IC_50_ (μM)
cisPt	MDA-MB-231 [59]	17 ± 4
	MDB-MB-453 [60]	10 ± 1
	H1299 [61]	25 ± 2
	DU145 [62]	4 ± 1
	RWPE-1 [63]	4 ± 1
l-argPt	MDA-MB-231 [59]	73 ± 10
	MDB-MB-453 [60]	35 ± 3
	H1299 [61]	73 ± 5
	DU145 [62]	12 ± 2
	RWPE-1 [63]	69 ± 5
l-ornPt	DU145 [62]	46 ± 12

To better understand the different activities between normal and prostate cancer cells, cellular accumulation of l-argPt and the less active l-ornPt was examined. Previously, whole cell cisPt uptake in cisPt-sensitive and cisPt-resistant lung tumor cells was determined using inductively coupled plasma mass spectrometry (ICP-MS) [64]. In that study, cisPt-resistant cancer cells had a four-fold lower uptake of cisPt compared to the cisPt-sensitive cells. The amount of cisPt coordinated to biological targets such as DNA and RNA has also been quantified using ICP-MS [65]. In the present study, cellular accumulation of AAPt in DU145 (cancer) and RWPE-1 (normal) human cell lines in comparison to cisPt was determined by ICP-MS. As shown in Table 2, accumulation of the platinum-based compounds varies depending on the cell line and compound identity. Consistent results were obtained when dilutions (2× and 10×) were carried out, followed by ICP-MS measurements and correction of the dilution factors (data not shown). The cisPt has an accumulation of 15 nmol per million cancer cells, which is 17-fold higher than its accumulation in normal cells. The l-ornPt and l-argPt have accumulations of 28 and 96 nmol per million cancer cells, respectively, which is 30-fold higher than the accumulation in normal cells for both AAPt compounds. These results show similar trends with the antiproliferative activities shown in Table 1 but also suggest varying mechanisms of uptake for AAPt and cisPt in the normal and cancer cell lines.

**Table 2:** Accumulation of cisPt and AAPt compounds in prostate cancer (DU145) and normal (RWPE-1) cells

Pt compounds	Cell lines	Accumulation (nmol/10^6^)
cisPt	RWPE-1	0.9 ± 0.3
	DU145	15 ± 8
l-ornPt	RWPE-1	0.9 ± 0.2
	DU145	28 ± 11
l-argPt	RWPE-1	3.2 ± 0.1
	DU145	96 ± 16

### Impact of platinum(II) complexes on DNA

The next goal of this study was to compare platination of DNA by the AAPt complexes and examine the impact of platination on DNA structure, more specifically DNA bending. We expanded our study to include both isomers of the AAPt complexes (*i.e*., D and L amino acids) and examined different DNA target sites containing dG or dA residues. The alteration of DNA structure (*e.g*., bending and unwinding) due to the presence of cisPt adducts has been documented by using a variety of methods [16, 19, 22, 66]. We were interested in examining the effects of the carrier ligands, which are typically amines, on DNA bending. In the present work, we explored the effects of arginine- and ornithine-based platinum complexes (AAPt) that have a stereocenter and (*N,O*)-type coordination in place of the typical diammine (*N,N*)-type coordination.

### Reactivity of AAPt complexes with DNA

Eight 22-nt DNA sequences (top and bottom strands) used in this study are shown in Fig. 2B. The 5′-phosphorylated top-strand DNAs were designed to have dG and dA residues in the middle of the sequence (*i.e*., d(GpG), d(ApG), d(GpA), or d(ApA)) with flanking dT and dC residues, such that platination would be favored at the middle purine dinucleotide site to generate a single bifunctional adduct, or one intrastrand cross-link, per two turns of the DNA helix. The bottom strands are complementary sequences suitable for annealing and generating duplex DNAs with two-nucleotide overhangs on the 3′ ends. The GG-containing duplex DNA was used previously for bending studies and was originally designed to avoid long runs of dA residues, which could cause undesired bending [43]. The overhangs allow for ligation to produce DNAs with lengths of 44, 66, 88, 110, etc., base pairs (bp). The monomer sequence length was optimal for inter-adduct distances in the oligomers to be equivalent to two helical turns of DNA after ligation. These ligated DNAs were then used to calculate the bend angles created by the platinum adducts.

Previous studies demonstrated that the oligonucleotide length is important for observing the DNA-bending effects [16]. DNAs of 22 bp were shown to have the correct phasing in which two full turns of DNA contained a single cisPt adduct in the middle of sequence. The same 22-bp DNA was selected for the current bending studies, but with changes to the middle nucleotides from GG to AG, GA, or AA, to accommodate the different sequence preferences of AAPt complexes (Fig. 2B). The purine sites indicated can form intrastrand cross-links with either cisPt or AAPt in their bisaquated forms. The amino acid-linked platinum(II) (AAPt) complexes used in this study (l-argPt, d-argPt, l-ornPt, and d-ornPt) are shown in Fig. 2A.

Freshly prepared activated cisPt/AAPt complexes (*i.e*., bisaquated species with the two chlorido ligands replaced by two aqua ligands) were incubated separately with the single-stranded DNA top strands. The unplatinated DNAs and platinated DNA products were separated on 20% denaturing polyacrylamide gels (shown in the Supplementary Information (SI), Figure S2). The gel images show good separation of the platinated DNAs and corresponding unplatinated DNAs. In the case of GG, AG, and GA, > 90% of the DNA reacted with cisPt, compared to 50% for the AA DNA. This result is consistent with previous studies in which N7 of dG was the preferred coordination site for cisPt [67]. The four DNAs show 30–50% conversion to the platinated species with the activated AAPt complexes (Figure S2).

The four platinated top-strand DNAs were isolated, desalted, and characterized by MALDI-TOF mass spectrometry (Figure S3 and Table S1). The MALDI mass spectra are consistent with the presence of a single platinum adduct for each DNA strand. These data support formation of bifunctional intrastrand cross-links with loss of two aqua ligands. Characterization of the slower mobility bands on the gels (see Figure S2) revealed multiple platination events, but these product DNAs were not explored any further.

### DNA ligations to reveal AAPt-induced bending

**Fig. 3:**
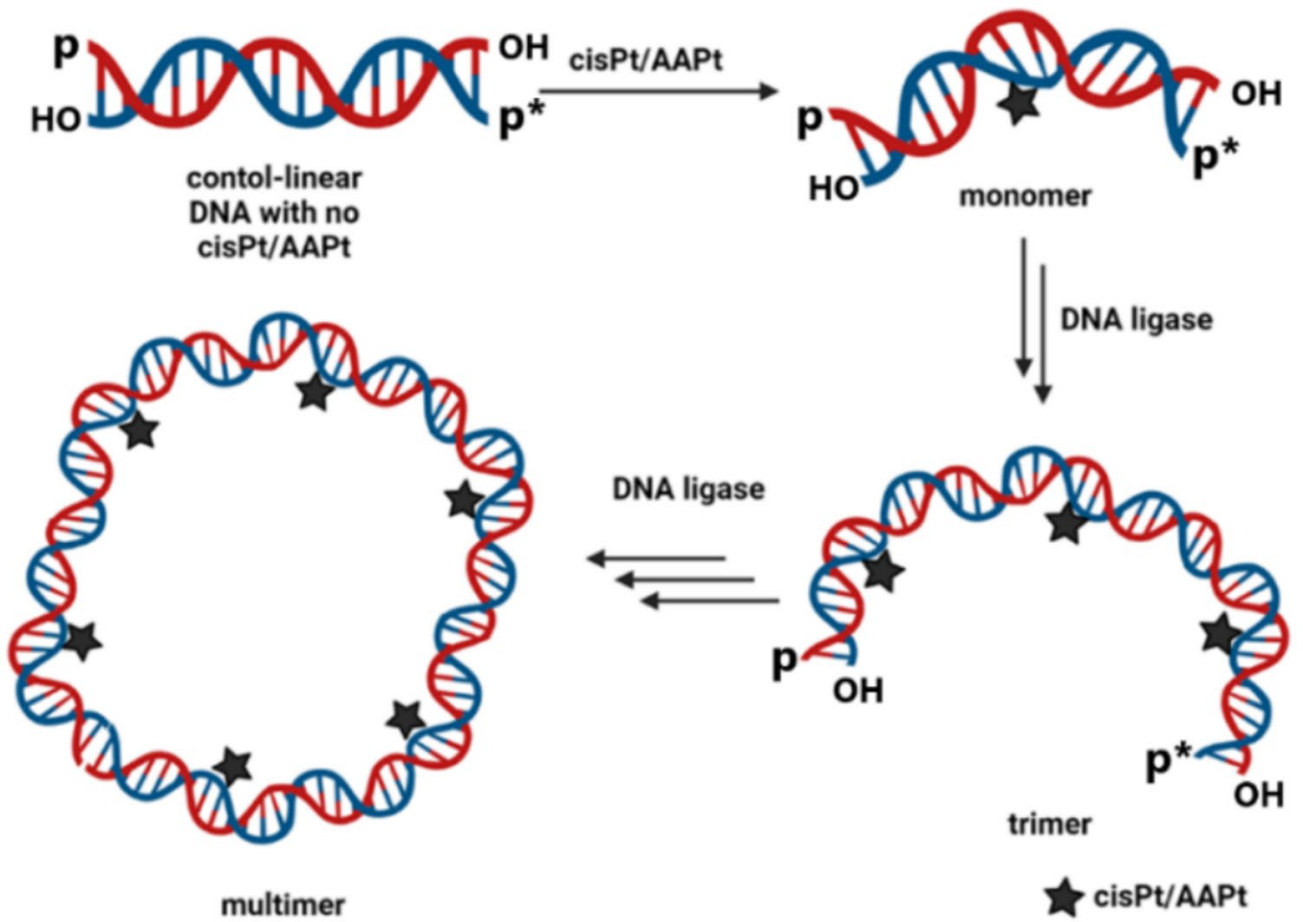
Generalized scheme for ligation of platinated DNA oligomers. Ligation of a 22-bp platinated DNA containing a bend in the helix and two-bp complementary overhanging ends leads to multimers with increased bending if the bends are phased constructively. The DNA product circularizes if the end-to-end distances are close due to the platinum-induced bending. The bottom strand (in blue) is radiolabelled with ^32^P (represented as p*)

The platinated 5′-phosphorylated top-strand DNAs were annealed to 5′-^32^P-labeled complementary bottom-strand DNAs and the resulting duplexes contained two-nt overhangs with hydroxyl groups on the 3′ ends. The overhangs are complementary such that the 22-bp DNAs can be ligated to form 44-, 66-, 88-, etc., bp DNAs as illustrated in Fig. 3. Control duplex DNAs were generated by annealing unplatinated top-strand DNAs with the complementary bottom strands. Following ligation of the 22-bp platinated DNAs, differences in gel mobilities were expected due the decreased end-to-end distances, or apparent shortening of the DNAs, as bending occurs [16]. With additional 22-bp units, constructively phased bends lead to a continued apparent shortening of the distances between the two DNA ends, which can be visualized by slower migration in a gel matrix [68]. As the DNA length increases and bending occurs, DNA circles also form at an optimal length and bend angle.

**Fig. 4:**
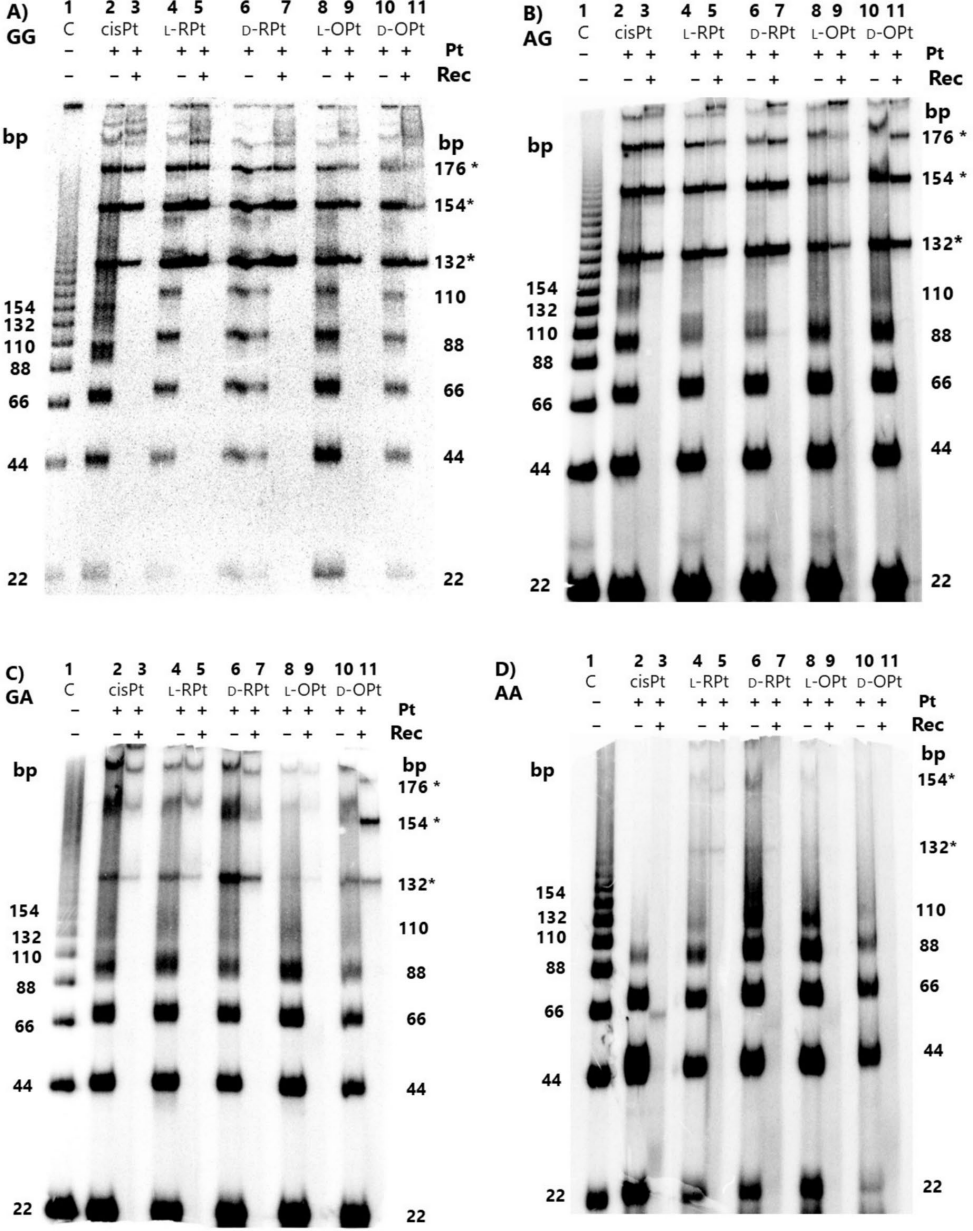
Autoradiograms of ligated products of double-stranded DNAs containing cisPt and AAPt adducts. Panels A-D contain GG, AG, GA, and AA DNAs, respectively. For each gel: lane 1 contains unplatinated DNA; lanes 2, 4, 6, 8, and 10 contain DNA with cisPt, l-argPt (l-RPt), d-argPt (d-RPt), l-ornPt (l-OPt), and d-ornPt (d-OPt), respectively; and lanes 3, 5, 7, 9, and 11 are the respective RecBCD-treated lanes to identify the circular DNA products. The DNAs were separated on 8% native polyacrylamide gels. The sizes of the ligated products are indicated on the right, with circular DNAs marked with an asterisk (*)

The oligomer products generated from treatment of the platinated DNAs (containing cisPt or AAPt bifunctional adducts) with T4 DNA ligase were separated on 8% native polyacrylamide gels, as shown in Fig. 4. Lane 1 of each gel contains unplatinated duplex DNA (control DNA), in which a ladder of monomer (22-bp) and multimer (44-, 66-, 88-, 110-bp) DNAs is observed. Similarly, the corresponding ligated platinated DNAs are observed as a ladder, but the DNAs display progressively slower mobilities compared to the control DNAs. A calibration curve was generated with the unplatinated DNA migration distances (SI, Figure S4), and the apparent lengths of the platinated DNAs were calculated. For all cases, the apparent lengths are higher than the actual lengths (*e.g*., the 44-bp GG DNA containing two cisPt adducts displays decreased mobility corresponding to a 49-bp DNA (Tables S2–S5)).

The slowed gel mobilities observed in Fig. 4 suggest decreased end-to-end distances of the platinated DNAs as the bending occurs, consistent with prior studies [16]. This difference in curvature of the platinated GG DNAs compared to the unplatinated DNAs is apparent for all platinum complexes tested, as shown in Fig. 4, panels A–D, lanes 2 (cisPt), 4 (l-argPt), 6 (d-argPt), 8 (l-ornPt), and 10 (d-ornPt). Similar behaviors occur with AG, GA, and AA DNAs, in which the platinated DNAs show slower mobilities than the corresponding unplatinated DNAs. The calibration curves for the AG, GA, and AA DNAs are also shown in Figure S4.

### Calculations of DNA bend angles

The bend angles for cisPt- and AAPt-containing DNAs were calculated using a previously developed empirical formula (Eq. 1) [68], in which R_L_ represents the relative mobility of each ligated DNA unit (from the 22-bp monomer), L is the length of the ligated product, and RC is the curvature of the DNA relative to an A_6_ tract DNA with a known bend angle (values given in Table S6) [44, 68].

1 $$R_{L} - 1 = \left( {9.6 \times 10^{ - 5} L^{2} - 0.47} \right)\left( {RC^{2} } \right)$$

**Fig. 5:**
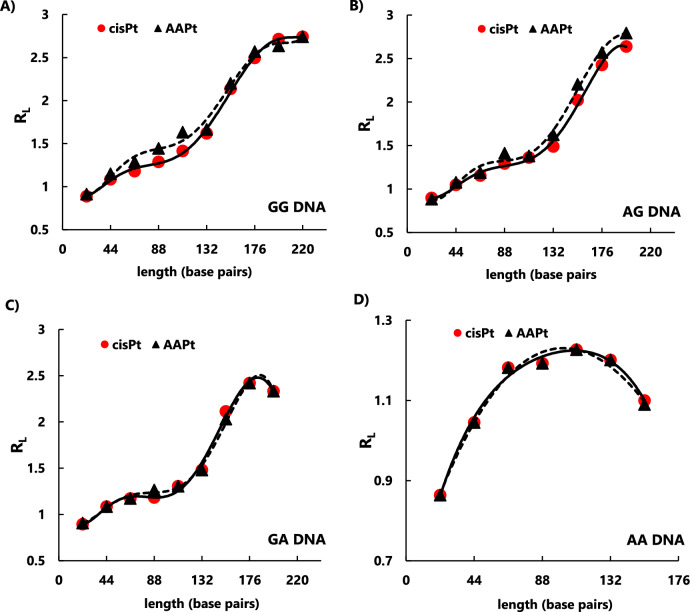
Plots of relative mobility for platinated GG, AG, GA, and AA DNAs. The calculated R_L_ values vs. actual lengths in base pairs are shown for the platinated, ligated DNAs: **A** GG-cisPt/AAPt, **B** AG-cisPt/AAPt, **C** GA-cisPt/AAPt, and **D** AA-cisPt/AAPt

The calibration curves from Figure S4 were used to calculate the *apparent length* of each platinated DNA multimer resulting from ligation (listed in Tables S2–S5). As mentioned earlier, the lengths of the platinated DNAs appear longer than the corresponding unplatinated DNAs. These values were used to calculate the *relative length* (or relative mobility), R_L_, for each product (R_L_ is the ratio of *apparent length* to the *actual length* of the DNA). The plots of R_L_ vs. length (base pairs) for each platinated DNA (GG, AG, GA, and AA) are shown in Fig. 5 (panels A–D, respectively). The R_L_ values for cisPt- and AAPt-modified DNAs are overlayed in each panel. Larger R_L_ values reflect slower movement of platinated multimer DNAs compared to the corresponding unplatinated DNAs due to a greater degree of bending. The plots are useful for comparing the level of bending for the four DNAs (GG, AG, GA, and AA) containing varying platinum adducts (cisPt or AAPt complexes). The DNAs containing the AAPt complexes all had the same mobilities regardless of the type (*i.e*., l-argPt, d-argPt, l-ornPt, or d-ornPt), so the R_L_ values in the plots are labeled as AAPt rather than individual complexes.

In Fig. 5, panel A, small differences in the R_L_ values are observed for the 66-, 88-, and 110-bp multimers of GG-cisPt and GG-AAPt, reflecting differences in DNA bending that become more apparent as the length, and also the number of platinum adducts, increases. The R_L_ values for the AG-cisPt and AG-AAPt DNAs are similar, with only slight deviations between the longer multimers (> 132 bp) (Fig. 5, panel B). The GA-cisPt and GA-AAPt DNAs both have similar R_L_ values as the AG-cisPt DNAs (Fig. 5, panel C) and do not show deviations between the platinum species. As with the AG and GA DNAs, the AA-cisPt and AA-AAPt DNAs display similar R_L_ values to one another (Fig. 5, panel D), but the plots have noticeable differences in the curvature compared to those of the GG, AG, and GA DNAs.

The differences in the plots for the platinated G-containing (*i.e*., GG, AG, GA) DNAs and AA DNA suggest that the sequence variations impact the phasing of the DNA bends or perhaps flexibility/unwinding of the DNA helix, as observed previously with differing types of DNA adducts (*e.g*., 1,2- vs. 1,3-adducts of cisPt) [16]. Another observation with the platinated GG, AG, and GA DNAs is the ability to form circular DNAs. Exonuclease V (RecBCD) treatment was used to verify the presence of circular DNAs. RecBCD cleaves linear DNAs, but the circular DNAs remain intact following enzyme digestion (Fig. 4, panels A–C, 132-bp DNA circles are indicated with an asterisk(*)) [69]. Circular DNAs are not observed with AA DNA with cisPt or ornPt adducts (Fig. 4, panel D), but the general gel patterns (*i.e*., formation of multimer species) are similar to those of the G-containing DNAs. A slight amount of the 132-bp circular product is observed for the AA DNA containing l/d-argPt adducts.

Equation 1 was used to determine the bend angles for platinated GG, AG, GA, and AA DNAs with cisPt or AAPt adducts. Following previous studies, the 110-bp platinated multimers were used to calculate the RC values (see Table S6) [16, 68]. The RC values represent the DNA curvature relative to an A_6_ tract. The absolute degree of curvature for the platinated DNA is determined by multiplying the relative curvature, RC, by the absolute value of the A_6_ tract bend, which was reported to be 20° [68]. The RC values are listed in Table S6 of the SI and the calculated bend angles for the platinated GG, AG, GA, and AA DNAs are shown in Table 3.

**Table 3:** Sequence-dependent bend angles for platinated DNAs

	GG	AG	GA	AA
cisPt	30 ± 1°	28 ± 1°	28 ± 1°	22 ± 2°
AAPt	37 ± 1°	30 ± 1°	29 ± 1°	22 ± 2°

The reduced mobility of multimers of GG-AAPt was more apparent compared to GG-cisPt (Fig. 4, panel A). The difference in position (*i.e*., gel mobility) of the multimers (see for example bands corresponding to 66-, 88-, and 110-bp DNAs) is clearly different for the GG-cisPt and GG-AAPt DNAs. These differences correspond to bend angles of 30 ± 1° for GG-cisPt and 37 ± 1° for the GG DNAs containing the four different amino acid-linked platinum adducts, summarized as GG-AAPt. The calculated bend angles for the platinated GG DNAs do not vary with the isomers (l-argPt and d-argPt; l-ornPt and d-ornPt). As in previous studies, the length at which the platinated DNA forms a circle was 132 bp [16], which we observed in this study for both cisplatin and the AAPts, regardless of the side chain (*i.e*., arg or orn) or stereochemistry (*i.e*., L or D).

The bend angles calculated for AG-cisPt and AG-AAPt are similar, 28 ± 1° and 30 ± 1°, respectively. These values are also in line with the bend angle for GG-cisPt, suggesting similar structural impacts for the platinum adducts and AG DNA. Although GA adducts with cisPt are observed less frequently in DNA, we were interested in determining the bend angles of GA DNAs containing either cisPt or AAPt adducts. The calculated bend angles for GA-cisPt and GA-AAPt are 28 ± 1° and 29 ± 1°, respectively, comparable to the value for GG-cisPt. Prior studies showed that AAPt complexes, specifically ornPt and argPt, have a preference for A residues [31, 70]; however, the AAPt complexes have similar ability as cisplatin to distort the AG and GA DNAs, with no apparent impact from the d or l isomers or different side chain structures. Thus, the ability to distort or bend the DNA does not seem to account for the sequence preferences of the platinum complexes.

Despite having similar calculated bend angles, the AG and GA DNAs do show some differences from the GG DNAs. In Fig. 4, panel A, a band corresponding to the 110-bp platinated GG DNA is clearly observed, followed by the more intense 132-bp circular DNA regardless of the type of platinum adduct. In contrast, the 110-bp bands are faint and less distinct for the platinated AG and GA DNAs. This difference could result from varying kinetics of formation of the 132-bp circular DNA, but further experiments are needed for verification. Larger circles of 154 and 176 bp are also observed for the platinated GG, AG, and GA DNAs, generated by ligation of seven and eight 22-bp monomer units, respectively. For the platinated GG, AG, and GA DNAs, the circular DNA intensities are higher than those of the linear DNAs, suggesting that the DNA is bending in phase and able to form circles on the timescale of the ligation reaction. This observation is most apparent for the GG DNAs with AAPt adducts, which were also determined to have the largest bend angles.

Platinum adducts of AA DNAs with cisPt and AAPt were also generated. The calculated bend angles of the AA DNAs are 22 ± 2° for both cisPt and AAPt adducts, based on the 110-bp DNA. As mentioned earlier, the gel patterns of the platinated AA DNAs differ from those of the GG, AG, and GA DNAs. There was no evidence for DNA circles with the AA DNAs containing cisPt or ornPt adducts (Fig. 4D), and only a slight amount of circular DNA products for the argPt-containing DNA. The smaller bend angle with AA DNA would lead to different phasing of the bends compared to the G-containing DNAs, and as such, a different length of the DNA would be needed to generate the circular DNAs. Alternatively, the AAPt adducts could be forming at different positions on the nucleobases (*i.e*., N7, N3, or N1 positions or various combinations of these positions), such that the structural impacts would also differ. Further high-resolution structure studies are needed to verify the sites of adduct formation for the A-containing DNAs and AAPt analogues.

## Conclusions

As determined by MTT assays, argPt exhibits similar antiproliferative activity as cisPt against certain cell lines (*e.g*., prostate cancer DU145). The l-argPt complex also has good selectivity for the prostate cancer line over the corresponding normal cell line RWPE-1 (IC_50_ values of 12 vs. 69 µM, respectively), whereas cisPt displays poor selectivity (identical IC_50_ values of 4 µM) for the same cell lines. The argPt also has a 30-fold higher accumulation in the prostate cancer cell line compared to the normal prostate cell line, which correlates well with the six-fold selective potency in cancer cells over normal cells. Further studies are necessary to determine the mechanism of uptake between the cell lines for the AAPt complexes. Follow-up studies will also include measuring antiproliferative activities and uptake of the d-argPt isomer, which showed identical impacts on DNA bending as the corresponding L isomer.

We characterized DNAs containing AAPt adducts to better understand AAPt reactivity and structural impacts on various target sequences. We chose small DNA model systems to characterize the reactivity and structural impacts of AAPt complexes. Previous work on 22-bp oligonucleotides revealed that cisPt-GG adducts induce DNA bending by 34° [16]. The 22-bp oligonucleotide is slightly more than two helical turns, such that upon adduct formation with cisPt, the DNA bends are in phase. Further evidence for in-phase bending is the presence of DNA circles, which can occur when the end-to-end distances of the ligated monomers decrease. If the bends are out of phase, the end-to-end distances would increase and decrease the likelihood for DNA circularization.

The AAPt complexes formed DNA adducts with all sequences tested (*i.e*., GG, AG, GA, and AA at the adduct site). We focused on purine-purine dinucleotide reactive sites, because prior reports showed GG as the major adduct formed with cisPt, and the GG-1,2-intrastand adducts are associated with greater activity of platinum(II) anticancer drugs [19, 71]. Both cisPt and AAPt display sequence-dependent bending, with decreasing bending in the following order: GG ≥ AG ≈ GA > AA for cisPt and GG > AG ≈ GA > AA for AAPt. In comparing bends angles of the GG DNA containing cisPt and AAPt adducts, we initially hypothesized that the seven-degree difference could be due to the bulkier, positively charged amino-acid side chains of arginine or ornithine compared to the smaller amine ligand in cisPt. Another contributing factor could be the (*N,N*)-type coordination of the amine ligands in cisPt versus (*N,O*)- or (*O,N*)-type coordination of the amino acid ligand in AAPt complexes. In addition, coordination with A residues could occur at the N7, N3, or N1 positions, which may also contribute to differences in the DNA bending [30]. Despite these differences between cisPt and AAPt, the similar bend angles observed for cisPt and AAPt adducts with the AG, GA, and AA DNAs suggest similar interactions of the ligands with the DNA. Furthermore, the adducts generated from isomers of AAPt complexes cause the same bend angles, revealing that the stereochemistry of the amino acid is not playing a significant role. A published X-ray structure of DNA containing an oxaliplatin GG adduct showed that there is space in the DNA major groove to accommodate a variety of ligand types and stereochemistries, although in that case a hydrogen bond between the (*R,R*)-DACH ligand and O6 of the 3′-G revealed the role of ligand chirality in mediating a specific interaction between the platinum complex and DNA [72].

We were interested in examining the role of chirality of the carrier ligand in AAPt complexes, since chirality at the alpha carbon of the amino acid could impact how the complex interacts with DNA. Previous studies showed that the flanking sequences surrounding the GG site had little impact on DNA bending for 1,2-GG adducts of cisPt [23]. In contrast, the DNA bend angles induced by 1,2-GG adducts of chiral complexes [Pt(DACH)]^2+^ and [Pt(DAB)]^2+^ were dependent on the flanking sequence [73]. In the case of AAPt complexes, the chirality had no impact on the DNA bend angle for the four DNA sequences tested when the flanking sequences were kept constant.

The different sequences of DNAs were also examined for their ability to form circles. If the bending caused by the platinum adduct was correctly phased, then DNA circles formed after ligation of six monomer units. The GG, AG, and GA DNAs containing cisPt or AAPt adducts formed circles of 132, 154, and 176 bp. In contrast, circularization of the platinated AA DNAs was minimal, with only the argPt-AA DNAs showing any formation of circular products. This lack of circular DNA formation was consistent with the smaller calculated bend angles for the AAPt DNAs and different the gel behaviors of the ligated products, suggesting different phasing of the overall DNA bends and longer end-to-end distances.

DNA bending can be induced by DNA-binding molecules and proteins [22, 43, 74]. DNA in its local environment is a flexible polymer with the potential to unwind, twist, and bend [75]. Adduct formation on DNA could lead to stabilization of those bends [16]. The adducts of cisPt and platinum(II) analogues reduce flexibility of the DNA because of the fixed square-planar geometry of the metal center [16, 22, 37]. The bending of the DNA might play roles in the antitumor mechanism of cisPt and other platinum(II)-based drugs. For example, the bent DNA could impact binding by proteins involved with DNA repair and either inhibit or facilitate this process [26, 76]. The platinum adducts may also hijack essential proteins away from their normal cellular functions [77]. Since the AAPt complexes have selective potency against some cancer cell lines, it will be of interest to explore possible interactions with DNA-binding proteins, and to examine protein binding to platinated DNAs with varying sequences and bend angles. The structural differences of the AAPt complexes from the perspective of chirality, side-chain identity, and mode of coordination (*i.e*., (*N,O*) vs. (*N,N*), orientation, N7/N3/N1 of A, etc.), could potentially impact biological processes such as replication and transcription in a different manner than cisPt, or alter resistance mechanisms. For future studies, biological, pharmacokinetic, AAPt uptake, and target selectivity would need to be carried out to relate the DNA structural impacts with clinical relevance.

A crystal structure of DNA containing an intrastrand cross-link of oxaliplatin with *R,R* stereochemistry [72] and differences in the DNA bend angles for the *R,R* and *S,S* isomers revealed the importance of ligand stereochemistry [40]. These structural differences of the isomers could also be related to the unique role of oxaliplatin and other platinum(II) analogues in mediating nucleolar stress and modes of action that differ from those of cisPt [78]. Therefore, deepening our understanding of novel cellular targets of platinum(II) analogues or varying modes of action are important for the future development of alternative metal-based anticancer compounds that might include AAPt complexes.

## Supplementary Information

Below is the link to the electronic supplementary material.

Supplementary file1 (PDF 1348 KB)

Supplementary file2 (PDF 7238 KB)

## Data Availability

Data (*e.g*., NMR spectra, mass spectra, gel images, and calibration curves) are provided within the manuscript and supplementary information files.

## Abbreviations

cisPt: *cis*-Diamminedichloridoplatinum(II), cisplatin
AAPt: Amino acid-linked platinum(II) complex
ornPt: Ornithine-linked platinum(II) complex
argPt: Arginine-linked platinum(II) complex
ICP-MS: Inductively coupled plasma mass spectrometry
