# Supplementary material for: Impacts of amino acid-linked platinum(II) complexes on DNA structure
Source: J Biol Inorg Chem. 2025 Jan 24;30(1):87–101. doi: 10.1007/s00775-025-02097-x (PMC11913917; doi:10.1007/s00775-025-02097-x)
Supplement: Supplementary file 1 — Supplementary file1 (PDF 1348 KB) [file 775_2025_2097_MOESM1_ESM.pdf]

Supplementary Information for

Impacts of amino acid-linked platinum complexes on DNA structure

Deepak Shrestha, Bett Kimutai, and Christine S. Chow*

Department of Chemistry, Wayne State University, Detroit, Michigan, USA

Table of Contents

**Figure S1**	NMR spectra of L and D isomers of argPt and ornPt complexes	pp. S2-S3
**Figure S2**	Polyacrylamide gel images of platinated top-strand oligonucleotides	p. S4
**Figure S3**	MALDI mass spectra of GG DNA control (unplatinated) and GG DNA with a cisplatin adduct	p. S5
**Figure S4**	Calibration curves for controls of four DNA sequences that were used to calculate the bend angles of platinated DNAs	p. S6
**Table S1**	MALDI mass data for platinated top-strand DNA sequences	p. S7
**Tables S2–S5**	Apparent length of oligonucleotide (base pairs) as compared to actual length (base pairs)	pp. S8-S9
**Table S6**	Relative curvature (RC) values for DNA bend angle calculations	p. S10

A) NMR spectra of arg, L-argPt, and D-argPt

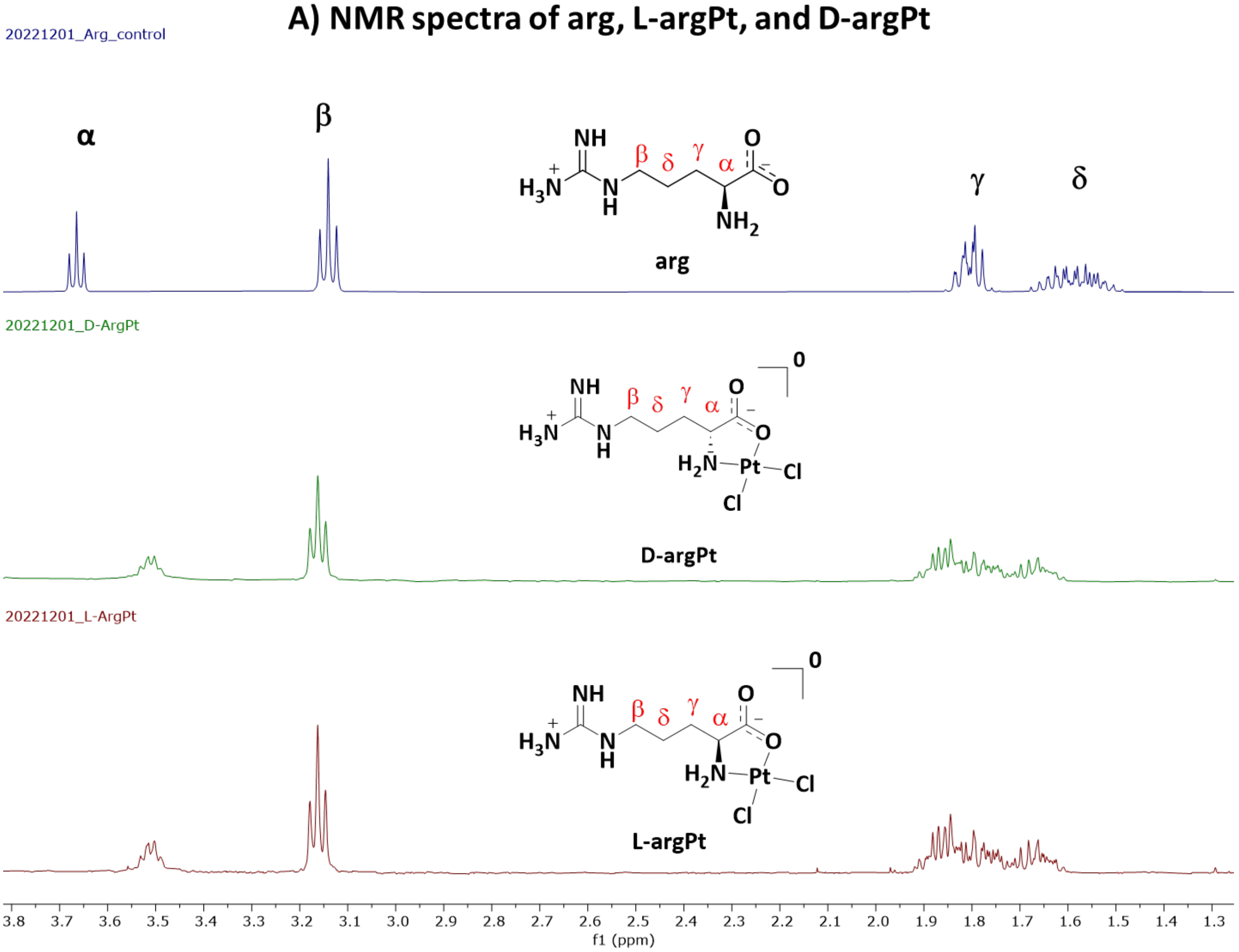

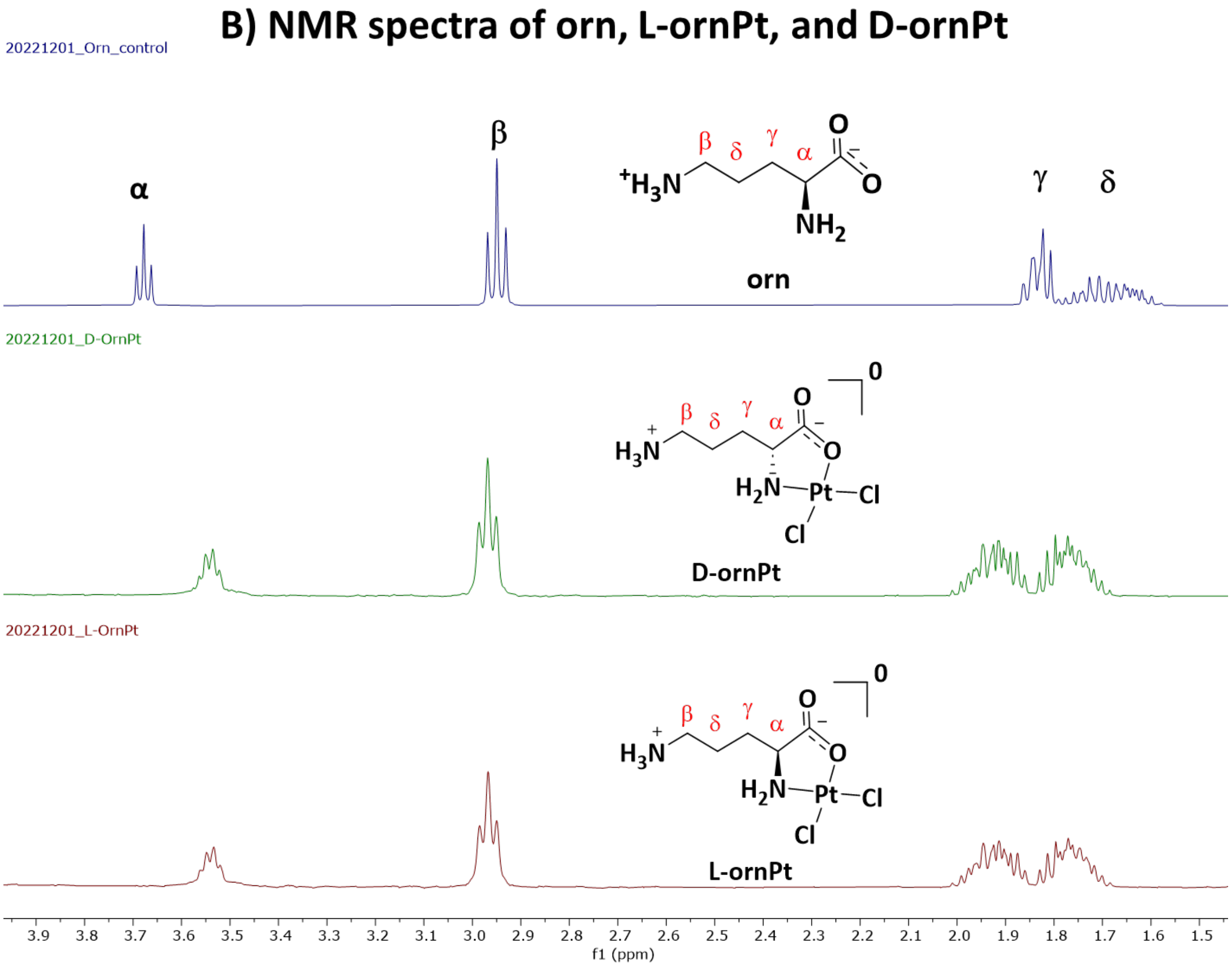

Figure S1: NMR spectra and respective AAPt structures. A) The arg (top), D-argPt (middle), and L-argPt (bottom) spectra are shown. B) The orn (top), D-ornPt (middle), and L-ornPt (bottom) spectra are shown. The NMR spectra of the D and L isomers of the AAPt complexes are the same, and the proton peaks shift upfield compared to amino acid controls (arg, orn) due to metal coordination.

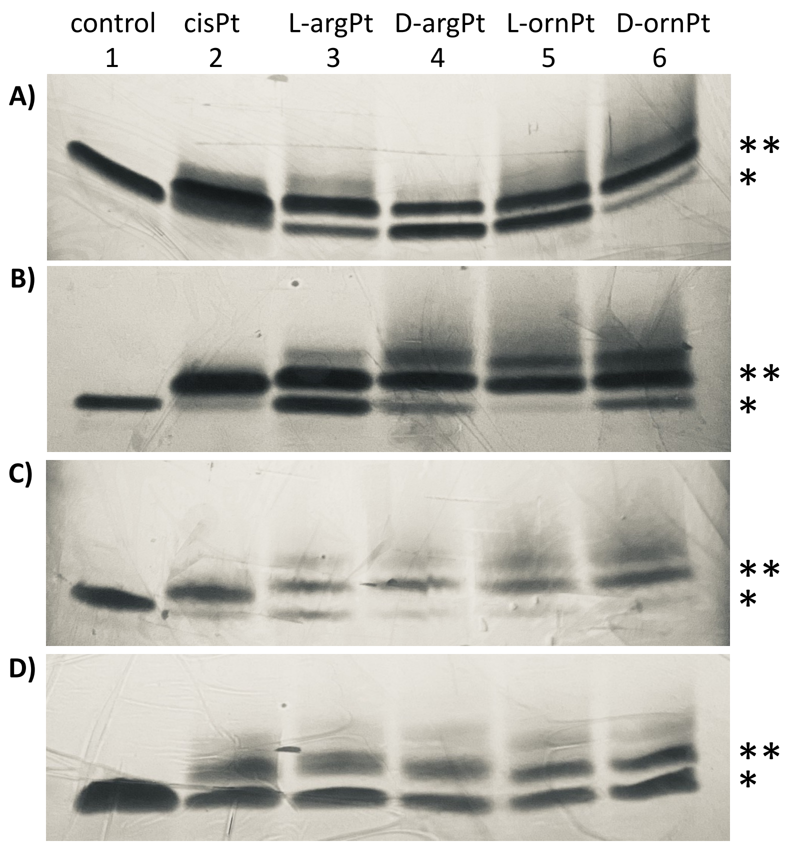

Figure S2: Separation of platinated DNAs on 20% denaturing polyacrylamide gels. The combinations of 22-nucleotide DNAs with aquated cisPt/AAPt complexes are as follows: A) GG; B) AG; C) GA; and D) AA with control DNA (no platinum), cisPt, L-argPt, D-argPt, L-ornPt, and D-ornPt in lanes 1 to 6, respectively. The faster mobility unplatinated (*) and slower mobility platinated (**) DNA bands are indicated.

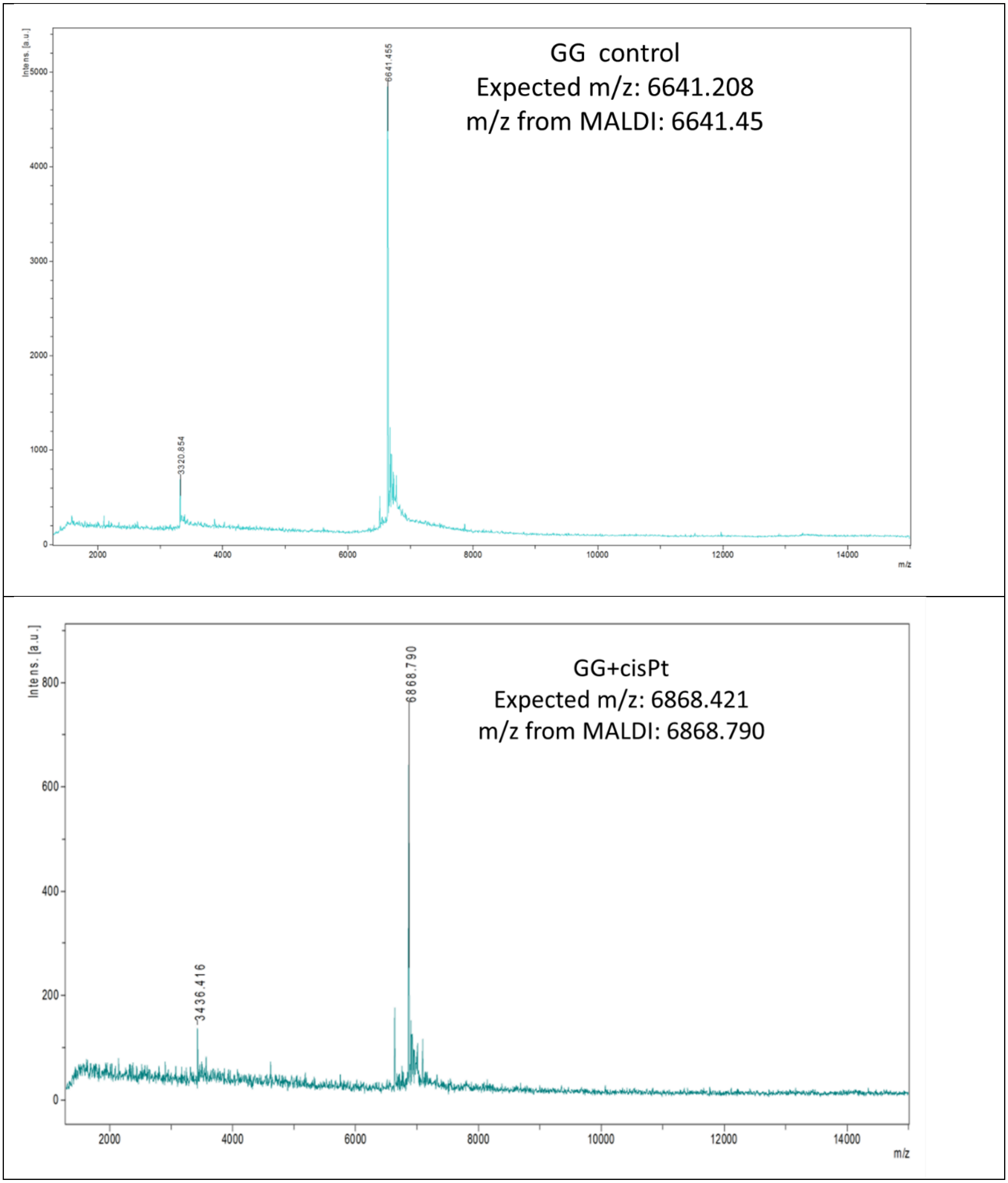

Figure S3: Representative MALDI mass spectra of unplatinated and platinated DNAs. The GG DNA control (no cisPt) (top) and platinated GG DNA (with cisPt adduct) are shown.

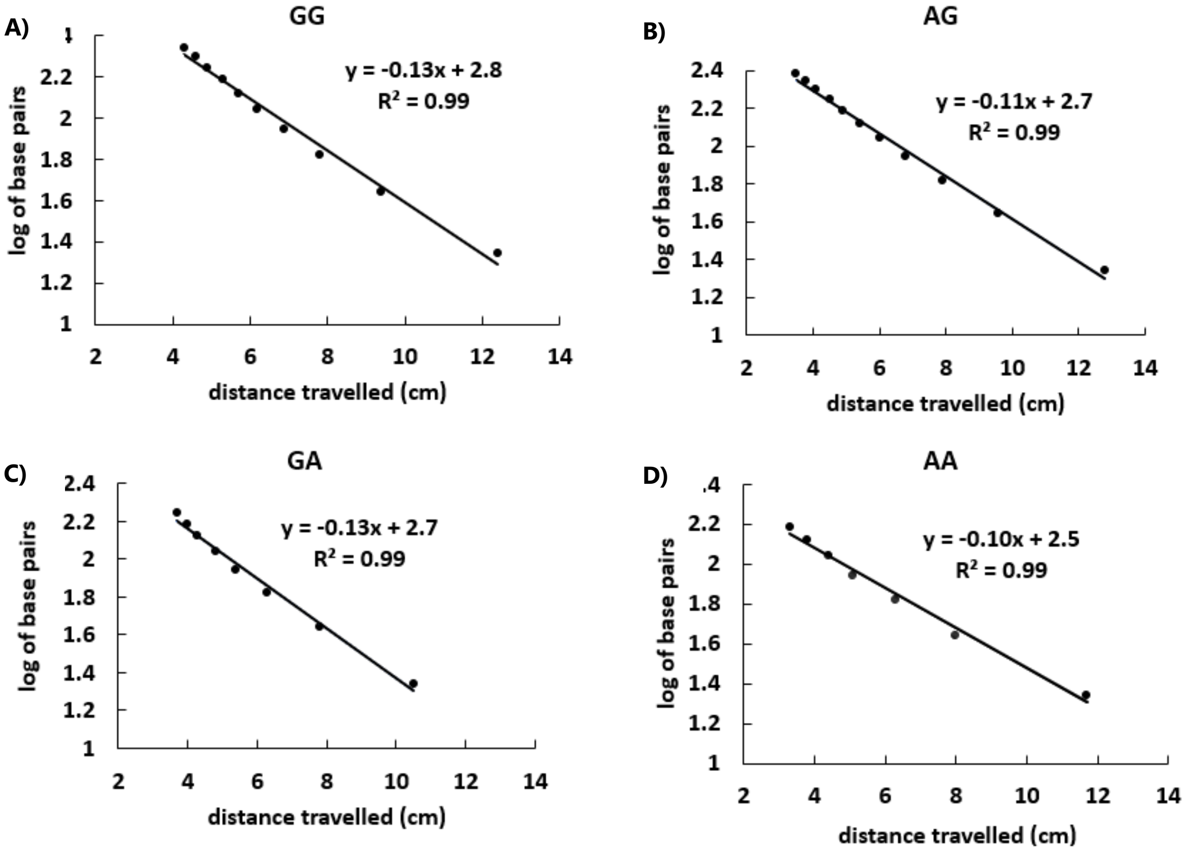

Figure S4: Calibration curves for unplatinated control DNAs. The plots of distance migrated vs. log of base pairs for ligated 22-nucleotide GG (A), AG (B), GA (C) and AA (D) DNAs are shown.

Table S1: MALDI mass data for top-strand DNAs with cisPt or AAPt adducts

Top strand DNA	Expected mass $[M+H]^+$	Experimental mass $[M+H]^+$
GG	6641.208	6641.450
GG+cisPt	6868.421	6868.790
GG+L-argPt	7008.561	7008.673
GG+D-argPt	7008.561	7008.673
GG+L-ornPt	6966.521	6966.731
GG+D-argPt	6966.521	6966.913
AG	6625.286	6625.903
AG+cisPt	6852.421	6852.386
AG+L-argPt	6992.561	6992.866
AG+D-argPt	6992.561	6992.341
AG+L-ornPt	6950.521	6951.122
AG+D-ornPt	6950.521	6950.724
GA	6625.286	6625.754
GA+cisPt	6852.421	6852.345
GA+L-argPt	6992.561	6992.646
GA+D-argPt	6992.561	6992.767
GA+L-ornPt	6950.521	6950.817
GA+D-ornPt	6950.521	6948.459
AA	6609.286	6609.400
AA+L-argPt	6976.561	6977.949
AA+D-argPt	6976.561	6976.173
AA+L-ornPt	6934.561	6934.417

Table S2: Actual vs calculated lengths of GG DNAs with Pt adducts

	Calculated lengths in base pairs (bp)				
Actual lengths in base pairs (bp)	cisPt	L-argPt	D-argPt	L-ornPt	D-ornPt
22	20	20	20	20	20
44	49	49	49	49	49
66	77	81	81	81	81
88	106	119	119	119	119
110	150*	165*	165*	165*	165*
132	203	203	203	203	203
154	305	305	305	305	305

*This value represents the length (in base pairs) used to calculate the DNA bend angle.

Table S3: Actual vs calculated lengths of AG DNAs with Pt adducts

	Calculated lengths in base pairs (bp)				
Actual lengths in base pairs (bp)	cisPt	L-argPt	D-argPt	L-ornPt	D-ornPt
22	20	20	20	20	20
44	48	48	48	48	48
66	77	81	81	81	81
88	108	114	114	114	114
110	148*	156*	156*	156*	156*
132	192	192	192	192	192
154	307	307	307	307	307
176	409	409	409	409	409

*This value represents the length (in base pairs) used to calculate the DNA bend angle.

Table S4: Actual vs calculated lengths of GA DNAs with Pt adducts

	Calculated lengths in base pairs (bp)				
**Actual lengths in base pairs (bp)**	**cisPt**	**L-argPt**	**D-argPt**	**L-ornPt**	**D-ornPt**
22	20	20	20	20	20
44	47	47	47	47	47
66	77	77	77	77	77
88	107	107	107	107	107
110	143*	143*	143*	143*	143*
110	196	196	196	196	196
132	318	318	318	318	318
154	418	418	418	418	418

*This value represents the length (in base pairs) used to calculate the DNA bend angle.

Table S5: Actual vs calculated lengths of AA DNAs with Pt adducts

	Calculated lengths in base pairs (bp)				
**Actual lengths in base pairs (bp)**	**cisPt**	**L-argPt**	**D-argPt**	**L-ornPt**	**D-ornPt**
22	19	19	19	19	19
44	46	46	46	46	46
66	78	78	78	78	78
88	105	105	105	105	105
110	135*	135*	135*	135*	135*
132	159	159	159	159	159

*This value represents the length (in base pairs) used to calculate the DNA bend angle.

Table S6: Relative curvature (RC) values based on distance travelled by ligated platinated DNAs (GG, AG, GA, and AA)

	RC values			
**Adduct type**	**GG**	**AG**	**GA**	**AA**
**cisPt**	0.75	0.70	0.60	0.55
**AAPt**	0.93	0.65	0.60	0.55
