# Supplementary material for: Impacts of amino acid-linked platinum(II) complexes on DNA structure
Source: J Biol Inorg Chem. 2025 Jan 24;30(1):87–101. doi: 10.1007/s00775-025-02097-x (PMC11913917; doi:10.1007/s00775-025-02097-x)

Supplementary Information for

Impacts of amino acid-linked platinum complexes on DNA structure

Deepak Shrestha, Bett Kimutai, and Christine S. Chow*

Department of Chemistry, Wayne State University, Detroit, Michigan, USA

Table of Contents

**Figure S1b**	Original uncropped gel images for Figure S2	p. S2b
**Figure S2b**	Original uncropped gel images for Figure 4	pp. S3b-S4b

Figure S5: Uncropped gel images for Figure S2

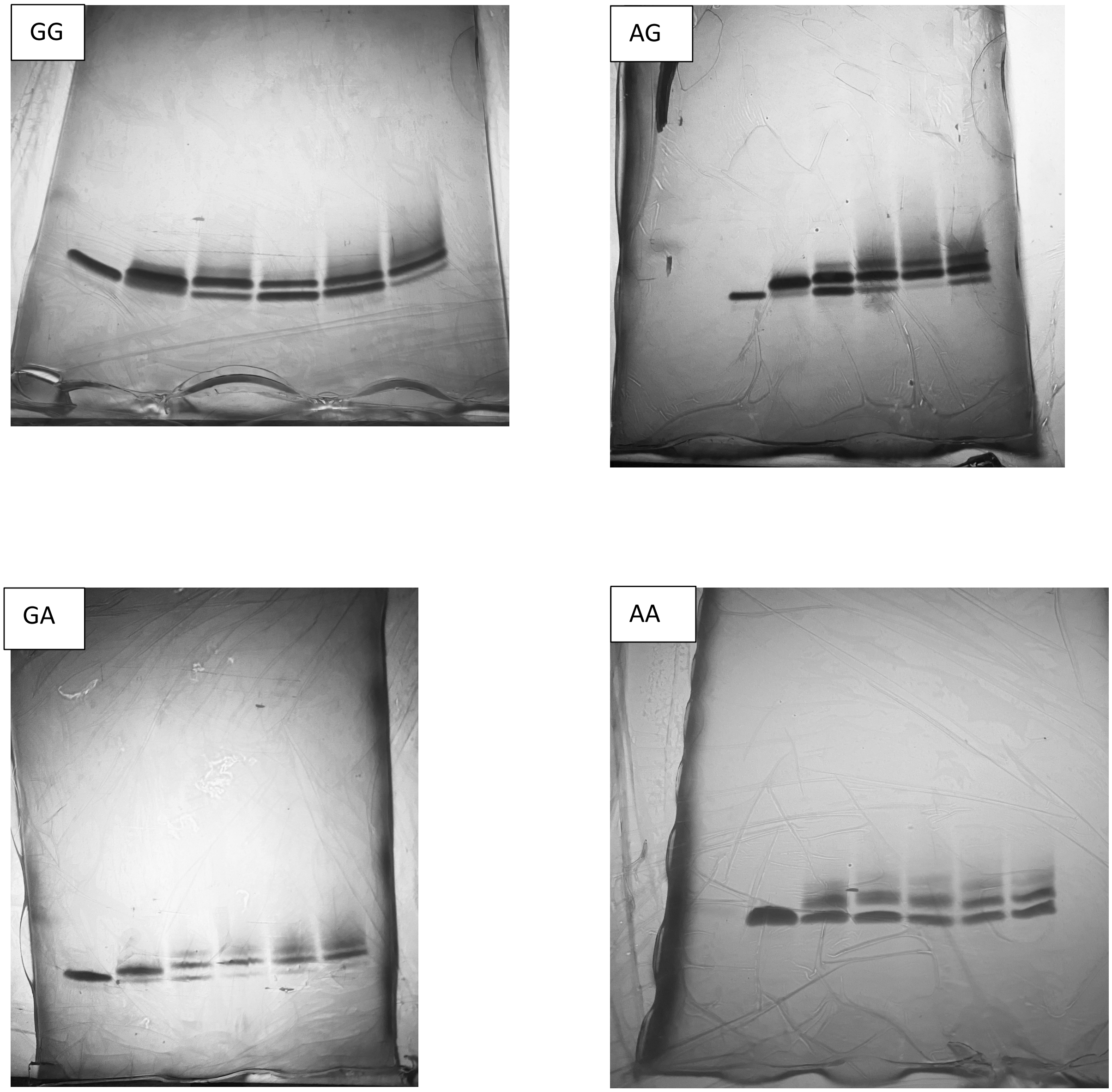

Figure S6. (A) DNA ligation gels from Figure 4 (upper left GG, upper right AG, lower left GA, lower right AA). Note: the entire gels are not shown because the gels are larger than the imaging screen; therefore, the wells are not visible for some gels. (B) An example of a full gel is given.

(A)

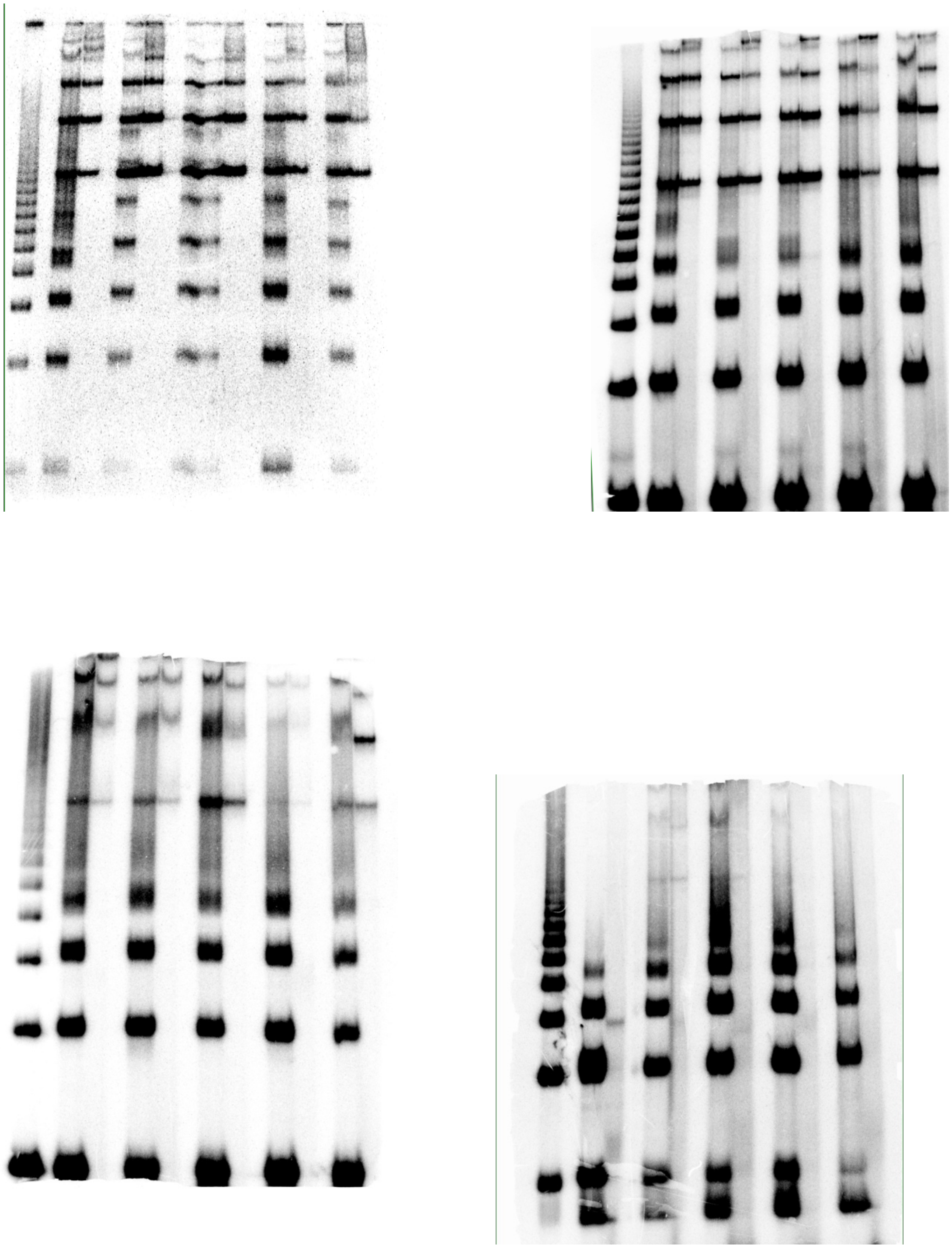

(B) **GG DNA**

these lanes are for a different experiment Con cisPt L-argPt D-argPt L-ornPt D-ornPt

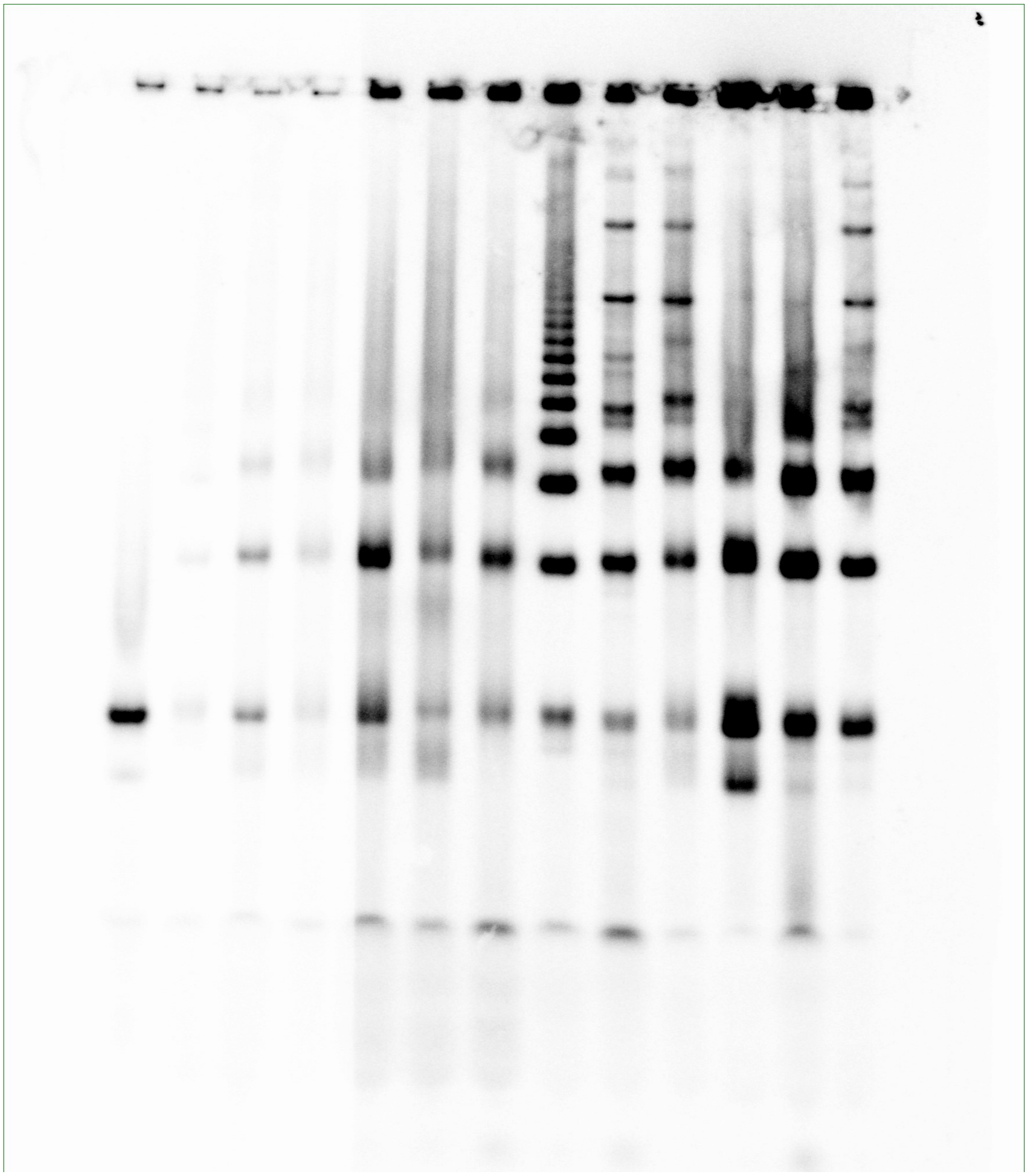

Supplement: Supplementary file 2 — Supplementary file2 (PDF 7238 KB) [file 775_2025_2097_MOESM2_ESM.pdf]
